# Calculation of arterial wall temperature in atherosclerotic arteries: effect of pulsatile flow, arterial geometry, and plaque structure

**DOI:** 10.1186/1475-925X-6-8

**Published:** 2007-03-01

**Authors:** Obdulia Ley, Taehong Kim

**Affiliations:** 1 Department of Mechanical Engineering, Texas A&M University, College Station, TX 77843, USA

## Abstract

**Background:**

This paper presents calculations of the temperature distribution in an atherosclerotic plaque experiencing an inflammatory process; it analyzes the presence of hot spots in the plaque region and their relationship to blood flow, arterial geometry, and inflammatory cell distribution. Determination of the plaque temperature has become an important topic because plaques showing a temperature inhomogeneity have a higher likelihood of rupture. As a result, monitoring plaque temperature and knowing the factors affecting it can help in the prevention of sudden rupture.

**Methods:**

The transient temperature profile in inflamed atherosclerotic plaques is calculated by solving an energy equation and the Navier-Stokes equations in 2D idealized arterial models of a bending artery and an arterial bifurcation. For obtaining the numerical solution, the commercial package COMSOL 3.2 was used. The calculations correspond to a parametric study where arterial type and size, as well as plaque geometry and composition, are varied. These calculations are used to analyze the contribution of different factors affecting arterial wall temperature measurements. The main factors considered are the metabolic heat production of inflammatory cells, atherosclerotic plaque length *l*_*p*_, inflammatory cell layer length *l*_*mp*_, and inflammatory cell layer thickness *d*_*mp*_.

**Results:**

The calculations indicate that the best location to perform the temperature measurement is at the back region of the plaque (0.5 ≤ *l*/*l*_*p *_≤ 0.7). The location of the maximum temperature, or hot spot, at the plaque surface can move during the cardiac cycle depending on the arterial geometry and is a direct result of the blood flow pattern. For the bending artery, the hot spot moves 0.6 millimeters along the longitudinal direction; for the arterial bifurcation, the hot spot is concentrated at a single location due to the flow recirculation observed at both ends of the plaque. Focusing on the thermal history of different points selected at the plaque surface, it is seen that during the cardiac cycle the temperature at a point located at *l*/*l*_*p *_= 0.7 can change between 0.5 and 0.1 degrees Celsius for the bending artery, while no significant variation is observed in the arterial bifurcation. Calculations performed for different values of inflammatory cell layer thickness *d*_*mp *_indicate the same behavior reported experimentally; that corresponds to an increase in the maximum temperature observed, which for the bending artery ranges from 0.6 to 2.0 degrees Celsius, for *d*_*mp *_= 25 and 100 micrometers, respectively.

**Conclusion:**

The results indicate that direct temperature measurements should be taken **(1) **as close as possible to the plaque/lumen surface, as the calculations show a significant drop in temperature within 120 micrometers from the plaque surface; **(2) **in the presence of blood flow, temperature measurement should be performed in the downstream edge of the plaque, as it shows higher temperature independently of the arterial geometry; and **(3) **it is necessary to perform measurements at a sampling rate that is higher than the cardiac cycle; the measurement should be extended through several cardiac cycles, as variations of up to 0.7 degrees Celsius were observed at *l*/*l*_*p *_= 0.7 for the bending artery.

## Background

Atherosclerosis refers to the thickening of the arterial wall; the lesion starts by adhesion and filtration of lipoproteins (LDL) and monocytes through the endothelium. The lesion grows as the monocytes are transformed into macrophages (inflammatory cells) and engulf LDL molecules to form foam cells. These factors, together with the migration of smooth muscle cells and the formation of cholesterol crystals, increase the arterial wall thickness which reduces the luminal area where the blood flow takes place.

A detailed description of the evolution, composition, and classification of atherosclerotic plaque can be found in [1,2]. As plaque grows and changes its geometry and composition, the thin fibrous cap atheroma is disrupted due to alterations in local shear stress. The fibrous cap then breaks, releasing the plaque contents into the arterial lumen. When underlying tissue is exposed, a thrombus is formed which obstructs blood flow and produces ischemic syndromes such as stroke, angina pectoris, and myocardial infarctions (MI).

Clinical and postmortem anatomical studies reveal that atherosclerotic lesions in humans develop preferentially along the inner wall of curved segments and the outer walls of bifurcations of relatively large arteries [3,4]. This is because the blood flow is disturbed by the occurrence of flow separation and formation of complex secondary and recirculation flows [5,6]. These sites correspond to locations where the fluid shear stress on the vessel wall is significantly lower in magnitude than the normal physiological value [8-10]. This pattern of plaque localization is observed regardless of diet and ethnicity [4].

Cardiovascular disease (CVD) is the third major cause of death in the developed world. In the United States, about 1.1 million patients annually suffer from acute myocardial infarction (AMI) [11] and nearly two thirds of the acute coronary syndromes pass undetected [12]. As a result, early detection and follow-up of vulnerable plaque is a major challenge that cardiologists face today because it is crucial in the prevention of acute cardiac events [13].

Strong interest in understanding atherosclerosis in the scientific community is indicated by the number of publications in the area, up from about 6,000 in the 1980's to 18,000 in 2000–2005. Modeling studies underlying the physical factors of cardiovascular disease and atherosclerosis are classified in the following main areas: **1) **the analysis of blood flow behavior to correlate between plaque location and wall shear stress [7,9,10]; **2) **the calculation of circumferential stress distribution in atherosclerotic vessels to analyze the hypothesized direct correlation between maximal stress and plaque rupture [14,15]; **3) **the analysis of the transport and accumulation of macromolecules, such as LDL and albumin, on the arterial wall to explain deposit formation in terms of local wall shear stress variations [8,16]; and **4) **the study of the endothelial transport of inflammatory cells or transmigration, using confocal laser microscopy [17-19] and chemotaxis [20].

The fact that atherosclerosis is recognized as an inflammatory disease, along with the correlation observed between plaque temperature and rupture likelihood, is awakening interest in the determination of the arterial wall temperature (AWT) and the characterization of plaque vulnerability through local temperature [21]. It is reported [22] that plaques have several regions with surface temperatures that vary reproducibly by 0.2–0.3°*C*, and 37% have substantially warmer regions with temperature variations between 0.4 and 2.2°*C*. Such hot spots appear in plaques due to the increased transport and activation of macrophages or inflammatory cells [1]. These penetrate the arterial wall, form a thin layer, and (due to their high metabolic activity) produce the observed thermal inhomogeneity or hot spot.

This paper demonstrates calculations of inflamed atherosclerotic plaque temperature profiles. These calculations reveal information about unstable plaques and are used to correlate plaque geometry, activity, and evolutionary stage to temperature profiles that can be measured using novel catheters [13]. These calculations are used to study the factors affecting the arterial wall temperature measurements taken using such catheters [12,22-24]. The information that can be extracted from the model and calculations presented here is: the best location at which to perform the measurement, an adequate measurement sampling rate in relation to cardiac cycle, and the cooling effect of blood flow during measurement. Information related to temperature gradients calculated with the model proposed herein can serve clinical research groups studying AWT [23,24] because there are divergent opinions about the cooling effect of blood flow and other parameters which affect direct temperature measurement.

The numerical determination of arterial wall temperature distribution is necessary in order to: **1) **observe the effect of vessel geometry; **2) **perform parametric studies rapidly; **3) **reduce animal experimentation; and **4) **improve measurement strategies involving AWT contact methods. In this paper, the arterial wall temperature distribution is calculated by solving the Navier-Stokes equations and the energy equation in the arterial lumen and arterial wall. In the plaque, a heat generation term (due to the metabolic heat released by the macrophages or inflammatory cells) is considered.

**Figure 1:**
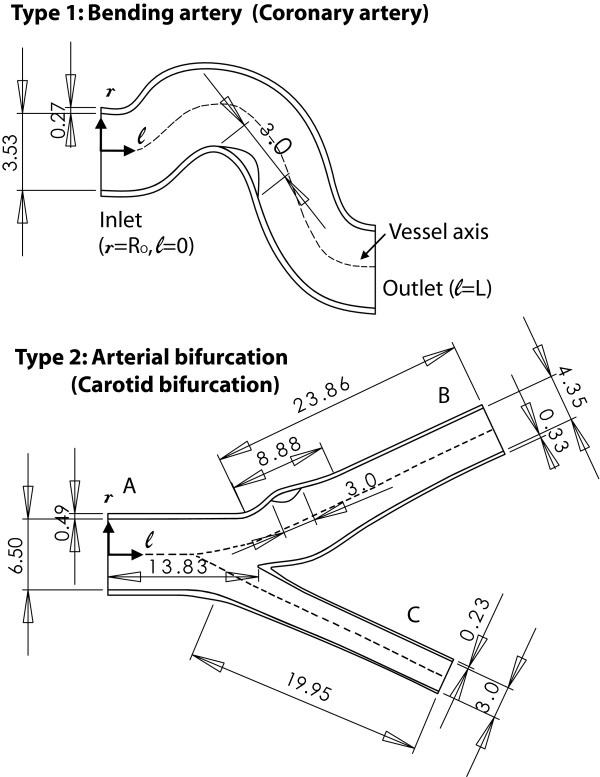
Vessel geometries considered and corresponding geometrical parameters. The dimensions presented are shown in millimeters.

For the calculations presented here, two different arteries which are known to commonly develop atherosclerotic plaques are considered: an arterial bend and an arterial bifurcation, with geometries and dimensions corresponding to a coronary artery and to a carotid bifurcation, respectively. Herein, 2D arterial models are considered in the longitudinal vessel direction. Such geometries are adopted based on previous studies dealing with mass transport [25-27] and stress concentrations in atherosclerotic plaques [28-30]; all of which provide useful physical insight and enable the reproduction of qualitative features while reducing the computational work/costs of three-dimensional (3D) models. The shape of the bending artery is obtained from an anatomically realistic arterial model [6] based on a photograph of a human coronary artery used in a previous flow study [31]. The geometry of the bifurcation artery is obtained from studies of a human carotid artery in [32,33]. The geometry types and dimensions are illustrated in Figure 1.

Recently, a similar study dealing with the estimation of plaque temperature has been published [34]. The present work, however, considers detailed plaque structures based on anatomical descriptions of atherosclerotic deposits [1]. Consideration of anatomically correct plaques is necessary in order to observe the combined effect of the convective cooling associated with blood flow as well as the metabolic production in the macrophage layer.

## Methods

### Blood flow in arteries

In this study, blood is treated as a homogeneous and incompressible Newtonian fluid. These assumptions are justified for the large arteries (where the blood components are small compared with the vessel diameter) and are also based on studies showing that the use of different viscosity models has only small effects on resulting blood flow [33,35]. Fluid-Structure interaction (FSI) is also neglected as recent studies indicate that neglecting the elastic nature of the arterial wall incurs an error of approximately 10% in the velocity profiles [36-38].

The transient calculations of arterial wall temperature and blood flow are carried out under the different physiological flows for the two arteries selected (bending artery and arterial bifurcation), but the vessel geometry and waveform effects of blood flows are not tested independently. The pulsatile blood flow is governed by the Navier-Stokes equations and the continuity equation for an incompressible fluid:

ρ(∂v∂t+v⋅∇v)=−∇P+μ∇2v,     (1)
 MathType@MTEF@5@5@+=feaafiart1ev1aaatCvAUfKttLearuWrP9MDH5MBPbIqV92AaeXatLxBI9gBaebbnrfifHhDYfgasaacH8akY=wiFfYdH8Gipec8Eeeu0xXdbba9frFj0=OqFfea0dXdd9vqai=hGuQ8kuc9pgc9s8qqaq=dirpe0xb9q8qiLsFr0=vr0=vr0dc8meaabaqaciaacaGaaeqabaqabeGadaaakeaaiiGacqWFbpGCdaqadaqaamaalaaabaGaeyOaIylcbeGae4NDayhabaGaeyOaIyRaemiDaqhaaiabgUcaRiab+zha2jabgwSixlabgEGirlab+zha2bGaayjkaiaawMcaaiabg2da9iabgkHiTiabgEGirlabdcfaqjabgUcaRiab=X7aTjabgEGirpaaCaaaleqabaGaeGOmaidaaOGae4NDayNaeiilaWIaaCzcaiaaxMaadaqadaqaaiabigdaXaGaayjkaiaawMcaaaaa@4D46@

**Figure 2:**
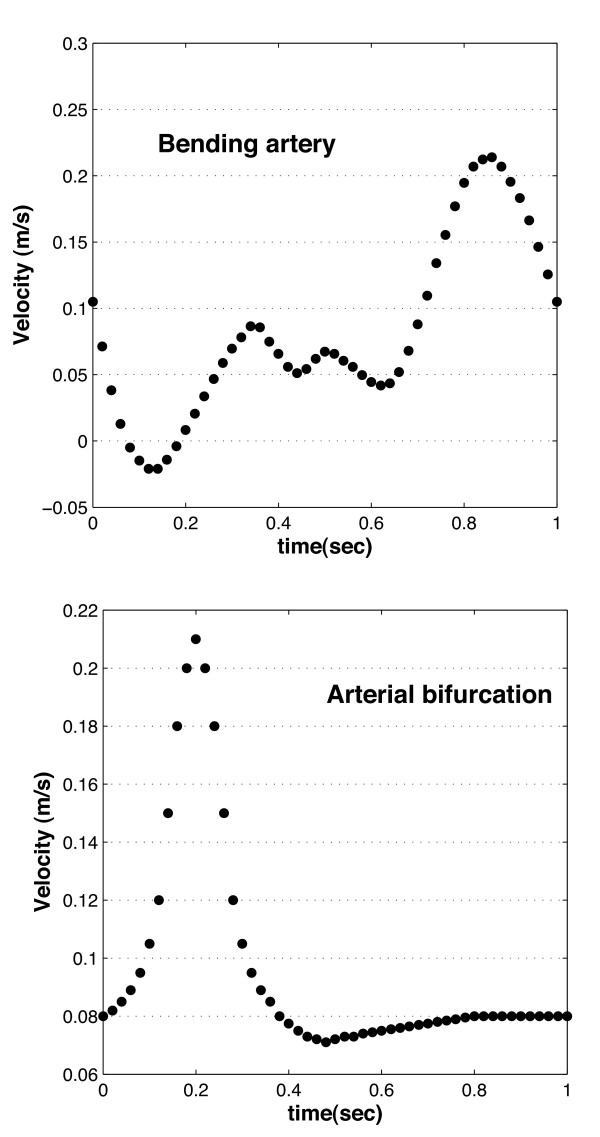
Pulsatile flow waveform used at the inlet of **(a) **the bending artery [35] and **(b) **the arterial bifurcation [41] [42].

∇·**v **= **0**,     (2)

where **v **is the blood velocity vector, *t *the time, *P *the driving pressure difference in the arterial segment, *ρ *the density of blood, and *μ *the viscosity of blood. A cartesian coordinate system is positioned at the center of the vessel entrance (*r *= 0). The presence of a water transmural flow inside the arterial wall is neglected in the calculations because it is very small in comparison to the blood flow in the luminal region. Details about common boundary conditions for the flow in arteries are widely available in the literature [3,7,38-40]. In this work, the inlet velocity profiles with the waveform function given in Figures 2a and 2b are used, where a heart rate of approximately 60 beats per minute is considered. These velocity profiles correspond to the pulsatile velocity profile in the right coronary artery (Figure 2a) of a a healthy 56-year-old female [35] and the profile of the carotid bifurcation [41,42] (Figure 2b).

### Temperature prediction at the arterial wall

Given the relationship between plaque vulnerability and local temperature, this paper shows calculations of temperature fields in large arteries containing small active plaques. To determine the temperature of the arterial wall and inflamed plaque, the atherosclerotic artery is modeled along the longitudinal direction. In this system, convection due to the luminal blood flow and heat conduction through the vessel walls are present, and the energy equation for the lumen and arterial wall regions takes the following forms

ρiCPi(∂Ti∂t)−∇⋅(ki∇Ti)=q˙mi,     (3)
 MathType@MTEF@5@5@+=feaafiart1ev1aaatCvAUfKttLearuWrP9MDH5MBPbIqV92AaeXatLxBI9gBaebbnrfifHhDYfgasaacH8akY=wiFfYdH8Gipec8Eeeu0xXdbba9frFj0=OqFfea0dXdd9vqai=hGuQ8kuc9pgc9s8qqaq=dirpe0xb9q8qiLsFr0=vr0=vr0dc8meaabaqaciaacaGaaeqabaqabeGadaaakeaaiiGacqWFbpGCdaWgaaWcbaGaemyAaKgabeaakiabdoeadnaaBaaaleaacqWGqbaucqWGPbqAaeqaaOWaaeWaaeaadaWcaaqaaiabgkGi2kabdsfaunaaBaaaleaacqWGPbqAaeqaaaGcbaGaeyOaIyRaemiDaqhaaaGaayjkaiaawMcaaiabgkHiTiabgEGirlabgwSixlabcIcaOiabdUgaRnaaBaaaleaacqWGPbqAaeqaaOGaey4bIeTaemivaq1aaSbaaSqaaiabdMgaPbqabaGccqGGPaqkcqGH9aqpcuWGXbqCgaGaamaaBaaaleaacqWGTbqBcqWGPbqAaeqaaOGaeiilaWIaaCzcaiaaxMaadaqadaqaaiabiodaZaGaayjkaiaawMcaaaaa@541B@

ρbCPb(∂Tb∂t+(v⋅∇)Tb)−∇⋅(kb∇Tb)=0,     (4)
 MathType@MTEF@5@5@+=feaafiart1ev1aaatCvAUfKttLearuWrP9MDH5MBPbIqV92AaeXatLxBI9gBaebbnrfifHhDYfgasaacH8akY=wiFfYdH8Gipec8Eeeu0xXdbba9frFj0=OqFfea0dXdd9vqai=hGuQ8kuc9pgc9s8qqaq=dirpe0xb9q8qiLsFr0=vr0=vr0dc8meaabaqaciaacaGaaeqabaqabeGadaaakeaaiiGacqWFbpGCdaWgaaWcbaGaemOyaigabeaakiabdoeadnaaBaaaleaacqWGqbaucqWGIbGyaeqaaOWaaeWaaeaadaWcaaqaaiabgkGi2kabdsfaunaaBaaaleaacqWGIbGyaeqaaaGcbaGaeyOaIyRaemiDaqhaaiabgUcaRiabcIcaOGqabiab+zha2jabgwSixlabgEGirlabcMcaPiabdsfaunaaBaaaleaacqWGIbGyaeqaaaGccaGLOaGaayzkaaGaeyOeI0Iaey4bIeTaeyyXICTaeiikaGIaem4AaS2aaSbaaSqaaiabdkgaIbqabaGccqGHhis0cqWGubavdaWgaaWcbaGaemOyaigabeaakiabcMcaPiabg2da9iabicdaWiabcYcaSiaaxMaacaWLjaWaaeWaaeaacqaI0aanaiaawIcacaGLPaaaaaa@5AEF@

where the subscript *i *refers to the different tissue regions present in the system, which correspond to arterial wall, plaque, and macrophage layer. *T*_*i *_represents the temperature, *k*_*i *_the thermal conductivity, *ρ*_*i *_the density, and *C*_*Pi *_the specific heat. q˙
 MathType@MTEF@5@5@+=feaafiart1ev1aaatCvAUfKttLearuWrP9MDH5MBPbIqV92AaeXatLxBI9gBaebbnrfifHhDYfgasaacH8akY=wiFfYdH8Gipec8Eeeu0xXdbba9frFj0=OqFfea0dXdd9vqai=hGuQ8kuc9pgc9s8qqaq=dirpe0xb9q8qiLsFr0=vr0=vr0dc8meaabaqaciaacaGaaeqabaqabeGadaaakeaacuWGXbqCgaGaaaaa@2E20@_*mi *_represents the metabolic heat produced by the tissue, which is neglected for the normal arterial wall and considered non zero for the macrophage layer only. The subscript *b *refers to blood properties and quantities, and **v **is the velocity of blood in the lumen region. The metabolic heat produced by the macrophages accounts for the local inflammatory reaction present in the plaque. The values of the thermal parameters for the blood, arterial wall, and plaque can be found in the literature [43-45] and are given in Table 1.

**Table 1:** Thermophysical parameters of blood, arterial wall, plaque tissue and macrophage layer

	Blood	Arterial wall	Plaque	Macrophage layer
*k*_*i *_(*W/m*°*C*)	0.549	0.476	0.484	0.484
*ρ*_*i *_(*kg/m*^3^)	1050	1075	920	920
*Cp*_*i *_(*J/kg*°*C*)	4390	3490	4080	4080
*μ*_*i *_(*Pa*·*s*)	0.0033			

These parameters where taken from [43].

For the thermal model, it is assumed that different layers in the vessel wall (intima, media, and adventitia) have the same thermal properties. The boundary conditions used to solve the equations (3) and (4) correspond to: **(1) **fixed temperature at the external vessel wall to account for the blood perfusion at the *vasa vasorum *(*T *= *T*_*a*_); **(2) **fixed blood and tissue temperature at the vessel inlet (*T *= *T*_*a*_), and **(3) **no temperature gradient at the vessel outlet (∂T∂n
 MathType@MTEF@5@5@+=feaafiart1ev1aaatCvAUfKttLearuWrP9MDH5MBPbIqV92AaeXatLxBI9gBaebbnrfifHhDYfgasaacH8akY=wiFfYdH8Gipec8Eeeu0xXdbba9frFj0=OqFfea0dXdd9vqai=hGuQ8kuc9pgc9s8qqaq=dirpe0xb9q8qiLsFr0=vr0=vr0dc8meaabaqaciaacaGaaeqabaqabeGadaaakeaadaWcaaqaaiabgkGi2kabdsfaubqaaiabgkGi2kabd6gaUbaaaaa@321E@ = 0). *T*_*a *_is a constant that represents the arterial or core temperature and is assigned a value of 37.5°*C*. Finally, continuity of heat flux and temperature at the lumen/plaque interface, the plaque/vessel wall interface, and the plaque/macrophage layer interface is assumed.

The metabolic heat released by the inflamed plaque is a direct function of plaque composition and the developmental stage of the lesion. Macrophages are involved in all evolutionary stages of the lesion, but their activation and subsequent metabolic heat production varies in each stage. Macrophages embedded in atherosclerotic plaques are highly active as they are in the process of engulfing lipid molecules [2]. However, there are no reports of cellular metabolic heat *q*_*cell *_for active macrophages in atherosclerotic plaques. Other highly active cells are hepatocytes, for which microcalorimetry studies report a basal heat production of 300 pW per cell [46]. Microcalorimetry is commonly used to follow cell development and activation [47,48], and it has shown that the metabolic heat production of cells is dependent on their concentration in the sample. For macrophages, cell volume as well as oxygen consumption increase proportionally [49,50]. In this study, q˙
 MathType@MTEF@5@5@+=feaafiart1ev1aaatCvAUfKttLearuWrP9MDH5MBPbIqV92AaeXatLxBI9gBaebbnrfifHhDYfgasaacH8akY=wiFfYdH8Gipec8Eeeu0xXdbba9frFj0=OqFfea0dXdd9vqai=hGuQ8kuc9pgc9s8qqaq=dirpe0xb9q8qiLsFr0=vr0=vr0dc8meaabaqaciaacaGaaeqabaqabeGadaaakeaacuWGXbqCgaGaaaaa@2E20@_*m *_was chosen to produce a maximum temperature change comparable with the reported measurement [21,51]. For the calculations, three different values for the heat generation of the macrophage layer are used and they correspond to q˙
 MathType@MTEF@5@5@+=feaafiart1ev1aaatCvAUfKttLearuWrP9MDH5MBPbIqV92AaeXatLxBI9gBaebbnrfifHhDYfgasaacH8akY=wiFfYdH8Gipec8Eeeu0xXdbba9frFj0=OqFfea0dXdd9vqai=hGuQ8kuc9pgc9s8qqaq=dirpe0xb9q8qiLsFr0=vr0=vr0dc8meaabaqaciaacaGaaeqabaqabeGadaaakeaacuWGXbqCgaGaaaaa@2E20@_*m *_= 0.05,0.1 and 0.2 *W*/*mm*^3^, these values are higher than the reported values of macrophages located in the lung of rabbits; higher values are assumed to consider the continuous involvement of the macrophages in engulfing lipid substances inside plaque. The value of q˙
 MathType@MTEF@5@5@+=feaafiart1ev1aaatCvAUfKttLearuWrP9MDH5MBPbIqV92AaeXatLxBI9gBaebbnrfifHhDYfgasaacH8akY=wiFfYdH8Gipec8Eeeu0xXdbba9frFj0=OqFfea0dXdd9vqai=hGuQ8kuc9pgc9s8qqaq=dirpe0xb9q8qiLsFr0=vr0=vr0dc8meaabaqaciaacaGaaeqabaqabeGadaaakeaacuWGXbqCgaGaaaaa@2E20@_*m *_for the macrophage layer was approximated considering the cellular volume and metabolic heat production of a single cell by the following relationship

q˙m[Wmm3]=qcellVcell,     (5)
 MathType@MTEF@5@5@+=feaafiart1ev1aaatCvAUfKttLearuWrP9MDH5MBPbIqV92AaeXatLxBI9gBaebbnrfifHhDYfgasaacH8akY=wiFfYdH8Gipec8Eeeu0xXdbba9frFj0=OqFfea0dXdd9vqai=hGuQ8kuc9pgc9s8qqaq=dirpe0xb9q8qiLsFr0=vr0=vr0dc8meaabaqaciaacaGaaeqabaqabeGadaaakeaacuWGXbqCgaGaamaaBaaaleaacqWGTbqBaeqaaOWaamWaaeaadaWcaaqaaiabdEfaxbqaaiabd2gaTjabd2gaTnaaCaaaleqabaGaeG4mamdaaaaaaOGaay5waiaaw2faaiabg2da9maalaaabaGaemyCae3aaSbaaSqaaiabdogaJjabdwgaLjabdYgaSjabdYgaSbqabaaakeaacqWGwbGvdaWgaaWcbaGaem4yamMaemyzauMaemiBaWMaemiBaWgabeaaaaGccqGGSaalcaWLjaGaaCzcamaabmaabaGaeGynaudacaGLOaGaayzkaaaaaa@4A72@

where *V*_*cell *_is the volume of a single cell and *q*_*cell *_is the heat produced by a single cell. It is assumed that macrophages have a volume of 30 to 60 *μm*^3 ^and a cellular heat production *q*_*cell *_similar to that of hepatocytes.

### Plaque composition, size and distribution

**Figure 3:**
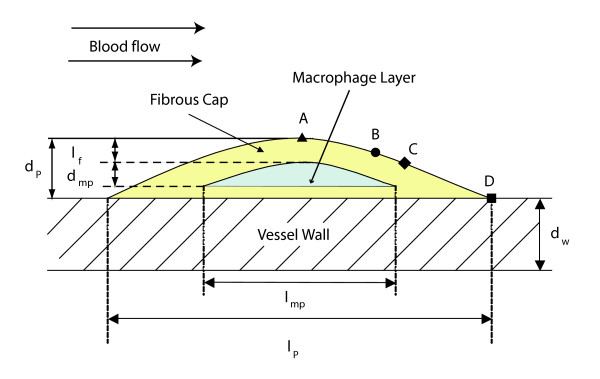
Plaque geometry and dimensions. *d*_*w *_is the arterial wall thickness, *d*_*p *_is the plaque thickness, *d*_*mp *_is the macrophage rich layer thickness, *l*_*f *_is the thickness of the fibrous cap. *l*_*p *_and *l*_*mp *_represent the extension or length of the plaque and the macrophage layer in the longitudinal direction, respectively. The location *x*_*i *_= *x*/*l*_*p *_of 4 observation points is presented. Point **A **corresponds to the center of the plaque (*x*_*A *_= 0.5), point **B **presents a point at *x*_*B *_= 0.65; point **C **is defined at *x*_*C *_= 0.7; and point **D **is the ending point of the plaque (*x*_*D *_= 1).

The temperature change in the vulnerable plaque is correlated to macrophage density and distribution, as well as the depth from the lumen surface at which the layer of macrophages are located. Vulnerable plaques showing thermal inhomogeneities of 0.4 to 2.2°*C *have a thickness of 400 *μm *and a macrophage rich layer between 15 to 40 *μm *thick [52]. In this study, the plaque size and the macrophage rich layer are described by Figure 3, where a longitudinal cross section of an atherosclerotic blood vessel containing a layer of macrophages is shown.

In Figure 3, the arterial wall thickness (*d*_*w*_) is set to be 5 to 10 percent of the vessel diameter. The plaque is located over the arterial wall, and its thickness (*d*_*p*_) is chosen to be *d*_*p *_= *αd*_*w*_, where *α *= 1, 2; these values of *α *represent the case of plaques that produce small occlusions and are difficult to observe with MRI or other contrast agent methods [53]. *l*_*f *_is the distance between the vessel lumen and macrophage layer, physically representing the fibrous cap thickness. Finally, *l*_*p *_and *l*_*mp *_denote the extension of the plaque and macrophage layer in the longitudinal direction of the vessel, respectively. The plaque is located in the region that corresponds to the lowest values of wall shear stress, and *l*_*p *_is extended to cover such a region in each one of the vessel types considered. The dimension *l*_*mp *_is selected to show high macrophage concentration at the center of the lesion [1]. Table 2 summarizes the dimensions of the arterial wall, plaque, and macrophage layer used in the calculations.

**Table 2:** Dimensions of plaque and macrophage (MC) layer used for bending artery and arterial bifurcation (unit: *μm*)

Vessel Type	Plaque length *l*_*p*_	Wall thickness *d*_*w*_	MC layer length *l*_*mp*_	Plaque thickness *d*_*p*_	Fibrous cap thickness *l*_*f*_	MC layer thickness *d*_*mp*_
Bending	3,000	270	1,500	540	25	50
					50	25
					50	50
					50	100
					100	50
Bifurcation	3,000	335	1,500	670	25	50
					50	25
					50	50
					50	100
					100	50

## Calculations

### Computations and mesh generation

Navier-Stokes equations and an energy equation for the two-dimensional (2D) transient case is solved using the multi-physics software COMSOL 3.2 (COMSOL Inc., SWEDEN). The software provides a complete FEA package with a unique equation-based modeling approach that allows for fully coupled multi-physics modeling in 2D and 3D. The FEM discretization of the time-dependent PDE problem produces a system of ordinary differential equations (ODE) or differential algebraic equations (DAE); COMSOL uses a version of the DAE solver DASPK [54] and a Newton-type iterative method.

The grid used is a non-structured mesh that is finer near the plaque and macrophage layer regions. The average number of triangular elements used in the calculation is 32,000. The number of elements used for the mesh is increased systematically to check for mesh independence; the mesh refinement is stopped when the maximum relative difference for the calculated parameter *P *for the different meshes satisfied the following condition

Pfine−PcoarsePfine≤0.1%;
 MathType@MTEF@5@5@+=feaafiart1ev1aaatCvAUfKttLearuWrP9MDH5MBPbIqV92AaeXatLxBI9gBaebbnrfifHhDYfgasaacH8akY=wiFfYdH8Gipec8Eeeu0xXdbba9frFj0=OqFfea0dXdd9vqai=hGuQ8kuc9pgc9s8qqaq=dirpe0xb9q8qiLsFr0=vr0=vr0dc8meaabaqaciaacaGaaeqabaqabeGadaaakeaadaWcaaqaaiabdcfaqnaaBaaaleaacqWGMbGzcqWGPbqAcqWGUbGBcqWGLbqzaeqaaOGaeyOeI0Iaemiuaa1aaSbaaSqaaiabdogaJjabd+gaVjabdggaHjabdkhaYjabdohaZjabdwgaLbqabaaakeaacqWGqbaudaWgaaWcbaGaemOzayMaemyAaKMaemOBa4MaemyzaugabeaaaaGccqGHKjYOcqaIWaamcqGGUaGlcqaIXaqmcqGGLaqjcqGG7aWoaaa@4B0D@

particularly, the maximum difference in the calculated temperature is 0.003% for the bending artery and 0.1% for the arterial bifurcation.

In addition to a grid independence analysis, a convergence verification for the initial condition is performed; this is needed to verify that the initial temperature used in the calculations corresponds to a stable temperature distribution. The calculations using the steady state temperature distribution are calculated using a velocity equal to the average velocity during one cardiac cycle (Fig. 2). Calculations are extended for several cycles using the waveform functions of Figure 2 as the inlet velocity condition; the number of cycles is selected to ensure that the temperature at several points along the plaque/lumen interface do not vary considerably between two consecutive cycles. For the arteries studied here, 6 cycles are necessary; the difference in the temperature distribution between the fifth and sixth cycles is 1.08% for the bending artery and 0.96% for the arterial bifurcation.

### Temperature calculations

In this study, emphasis is given to the characterization of the temperature inhomogeneity considering the following parameters: **1) **the arterial geometry and plaque location (Figure 1), **2) **the extension of the macrophage layer (*d*_*mp*_), and **3) **the heat generation produced by the macrophage layer (q˙
 MathType@MTEF@5@5@+=feaafiart1ev1aaatCvAUfKttLearuWrP9MDH5MBPbIqV92AaeXatLxBI9gBaebbnrfifHhDYfgasaacH8akY=wiFfYdH8Gipec8Eeeu0xXdbba9frFj0=OqFfea0dXdd9vqai=hGuQ8kuc9pgc9s8qqaq=dirpe0xb9q8qiLsFr0=vr0=vr0dc8meaabaqaciaacaGaaeqabaqabeGadaaakeaacuWGXbqCgaGaaaaa@2E20@_*m*_). This paper considers variations of *d*_*mp *_and q˙
 MathType@MTEF@5@5@+=feaafiart1ev1aaatCvAUfKttLearuWrP9MDH5MBPbIqV92AaeXatLxBI9gBaebbnrfifHhDYfgasaacH8akY=wiFfYdH8Gipec8Eeeu0xXdbba9frFj0=OqFfea0dXdd9vqai=hGuQ8kuc9pgc9s8qqaq=dirpe0xb9q8qiLsFr0=vr0=vr0dc8meaabaqaciaacaGaaeqabaqabeGadaaakeaacuWGXbqCgaGaaaaa@2E20@_*m *_during a cardiac cycle because steady state calculations indicate that these parameters have more influence over the plaque temperature [55].

The plots presented herein indicate the temperature along the plaque/lumen surface and its variation with time. In these plots, the horizontal axis coincides with the base of the plaque, and the temperature at the plaque/lumen interface is plotted with respect to its horizontal location. In order to compare the temperature distribution at the plaque/lumen interface of the different arteries studied, the horizontal position is divided by the plaque length (*l*_*p*_). The other type of plots presented here correspond to the time variation of the temperature in a selected point at the plaque/lumen surface.

As an initial condition of the transient calculation, the steady state solution is calculated with the uniform velocities of 0.08 *m/s *and 0.096 *m/s *for the bending artery and the arterial bifurcation, respectively. These values correspond to the average velocity during the cardiac cycle in each arterial geometry. The calculations are then performed for 6 cycles to attain a stationary state as discussed in the previous section.

**Figure 4:**
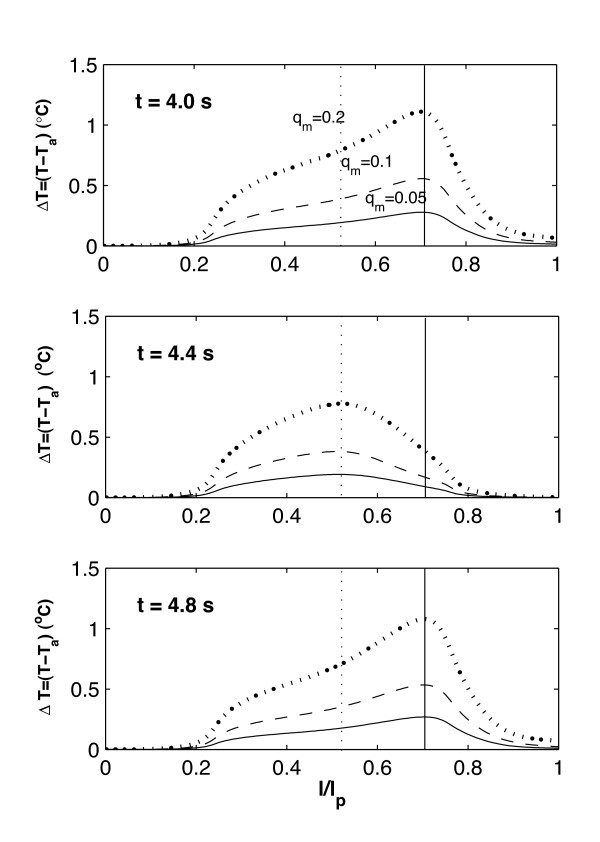
Transient temperature distribution at the plaque/lumen interface for the bending artery at different times, produced by variations in the local heat generation in the plaque q˙
 MathType@MTEF@5@5@+=feaafiart1ev1aaatCvAUfKttLearuWrP9MDH5MBPbIqV92AaeXatLxBI9gBaebbnrfifHhDYfgasaacH8akY=wiFfYdH8Gipec8Eeeu0xXdbba9frFj0=OqFfea0dXdd9vqai=hGuQ8kuc9pgc9s8qqaq=dirpe0xb9q8qiLsFr0=vr0=vr0dc8meaabaqaciaacaGaaeqabaqabeGadaaakeaacuWGXbqCgaGaaaaa@2E20@_*m *_(*W*/*mm*^3^). The times presented correspond to t = 4.0, 4.4 and 4.8 s. The inlet velocity profile for this arterial geometry is given in Figure 2a.

**Figure 5:**
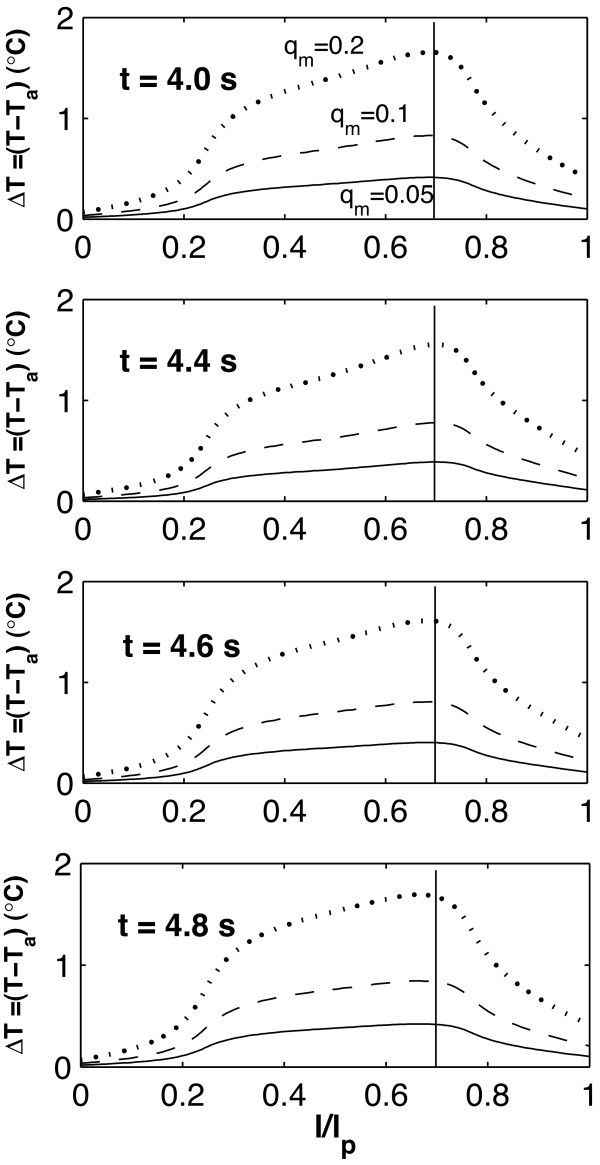
Transient temperature distribution at the plaque/lumen interface for the arterial bifurcation at different times. Temperature distribution is produced by variations in the local heat generation in the plaque q˙
 MathType@MTEF@5@5@+=feaafiart1ev1aaatCvAUfKttLearuWrP9MDH5MBPbIqV92AaeXatLxBI9gBaebbnrfifHhDYfgasaacH8akY=wiFfYdH8Gipec8Eeeu0xXdbba9frFj0=OqFfea0dXdd9vqai=hGuQ8kuc9pgc9s8qqaq=dirpe0xb9q8qiLsFr0=vr0=vr0dc8meaabaqaciaacaGaaeqabaqabeGadaaakeaacuWGXbqCgaGaaaaa@2E20@_*m*_(*W*/*mm*^3^). The times indicated correspond to *t *= 4.0,4.4,4.6 and 4.8 *s*. The inlet velocity profile for this arterial geometry is given in Figure 2b.

In the steady state calculations [55], it is observed that the magnitude and location of maximum temperature Δ*T*_*max *_is a function of the vessel geometry. In the transient case, it is seen that the magnitude and location of Δ*T*_*max *_is affected by the inlet waveform function as well as the vessel geometry. Figures 4 and 5 show the temperature distribution over the plaque surface (plaque/lumen interface) at different times through the cardiac cycle; the times selected are chosen to represent times where the velocity profile at the vessel entrance (Figure 2) shows a maximum, a minimum, or other meaningful behavior.

For the bending artery, the representative times are chosen as: **a) ***t *= 4.0 and 5.0 *s*, which represent the beginning and the end of the cardiac cycle; **b) ***t *= 4.4 *s*, where the peak of a small forward flow occurs; and **d) ***t *= 4.8 *s *or a time approximately halfway through the rapid forward flow. For the arterial bifurcation, on the other hand, the representative times selected correspond to *t *= 4.0,4.4,4.6, and 4.8 seconds. As seen in Figure 2b, the velocity waveform for the bifurcation does not present significant variation after *t *= 4.5 *s*; however, *t *= 4.4 *s *is chosen instead of *t *= 4.2 *s *because the pulse propagation of the extreme peak at *t *= 4.2 *s *reaches the plaque region at *t *= 4.4 *s *due to the distance between the vessel inlet and the plaque location. As observed in the steady-state case [55], as the blood velocity increases, the temperature at the plaque/lumen interface decreases and vice-versa.

## Results and discussion

Figure 4, shows a temporal temperature change of between 0.2°*C *and 1.1°*C *for *q*_*m *_= 0.05 *W*/*mm*^3 ^and *q*_*m *_= 0.2 *W*/*mm*^3 ^respectively. The maximum temperature peaks vary proportionally to the inlet blood flow, *i.e. *Δ*T*_*max *_is reduced at t = 4.4s and increases after *t *= 4.8 *s*. The maximum spatial temperature change during the cardiac cycle is located between 0.5 <*l*/*l*_*p *_< 0.7 (the solid line represents the location of Δ*T*_*max *_at *t *= 4.0 *s *and the dashed line indicates Δ*T*_*max *_at *t *= 4.4 *s*). It is observed that spatial Δ*T*_*max *_occurs closer to the center of the plaque when the blood velocity is reduced; it moves to the downstream edge of the plaque as blood flow increases in the last part of the cardiac cycle.

**Figure 6:**
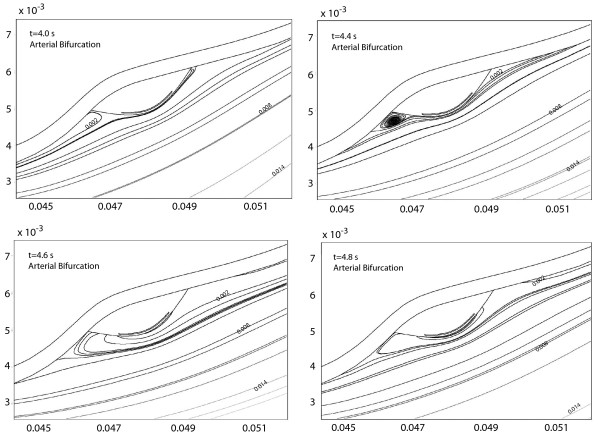
Flow circulation observed around an inflamed plaque located in an arterial bifurcation. Results shown correspond to calculations at times t = 4.0, 4.4, 4.6 and 4.8 s. The inlet velocity profile for this arterial geometry is given in Figure 2b.

**Figure 7:**
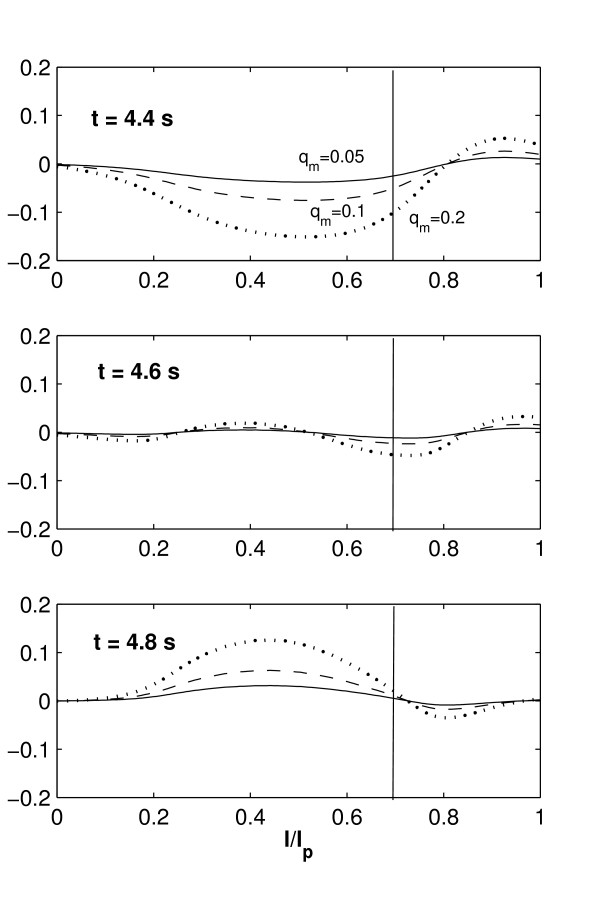
Plots of Δ*T*(*t*) - Δ*T*(*t *= 0) along the plaque/lumen interface for the arterial bifurcation at different times *t*, for various metabolic heat production values corresponding to q˙
 MathType@MTEF@5@5@+=feaafiart1ev1aaatCvAUfKttLearuWrP9MDH5MBPbIqV92AaeXatLxBI9gBaebbnrfifHhDYfgasaacH8akY=wiFfYdH8Gipec8Eeeu0xXdbba9frFj0=OqFfea0dXdd9vqai=hGuQ8kuc9pgc9s8qqaq=dirpe0xb9q8qiLsFr0=vr0=vr0dc8meaabaqaciaacaGaaeqabaqabeGadaaakeaacuWGXbqCgaGaaaaa@2E20@_*m *_= 0.2, 0.1 and 0.05 *W*/*mm*^3 ^and inlet velocity profile of Figure 2b. The times indicated correspond to the times *t *= 4.4,4.6 and 4.8 *s*.

In contrast to the bending artery, where the maximum spatial temperature changes its location with time, the maximum spatial temperature observed in the arterial bifurcation (Figure 5) does not drastically change its location (*l*/*l*_*p *_= 0.7) during the cardiac cycle. In spite of a significant peak of inlet pulsatile flow at *t *= 4.2 *s*, the temperature distribution in the downstream edge of the plaque remains almost unchanged; the temperature distribution at the front edge of the plaque presents slight variations with time resulting from the inlet flow. This is explained by the appearance of flow circulation at both anterior and posterior regions of the plaque. Flow circulation produces incompressible triangular cavities which prevented the cooling effect of blood flow and keep circular flow regions unable to transport thermal energy at the plaque surface. Figure 6 shows the stream lines with the appearance of flow circulation for times *t *= 4.0, 4.4, 4.6, and 4.8 *s*. To observe the temperature variations during the cardiac cycle, Figure 7 shows plots of Δ*T*(*t*) - Δ*T*(*t *= 0) along the plaque/lumen interface.

As in Figure 5, the vertical solid line indicates the location of spatial Δ*T*_*max *_at the beginning of the cycle (*t *= 4.0). It is seen that in the first part of the cycle (*t *< 4.6 *s*) the temperature at the front edge of the plaque drops due to convective cooling produced by an increase in blood flow velocity; as the blood velocity drops after *t *= 4.6 *s*, the temperature at the front edge of the plaque/lumen interface increases. The variation in the temperature distribution at the front edge of the plaque results from the flow circulation seen in Figure 6.

From Figures 4 and 5, three thermal regions are observed at the plaque/lumen interface and they are: **(1) **a front region where temperature is not affected by the presence of the macrophage layer and which changes according to the blood flow; **(2) **a middle region where temperature is directly affected by the macrophage layer dimensions and local convective heat transfer; and **(3) **a rear region where the convective heat transfer due to the blood flow is dominant over the presence of the macrophage layer. To study the regional variations of the plaque/lumen temperature, four points (**A**, **B**, **C **and **D**) are defined on the plaque lumen surface. Point **A **corresponds to the center of the plaque, point **B **presents the ending point of the middle region, point **C **is the beginning point of the rear region, and point **D **is the ending point of the plaque. The relative location of these points for the two arterial geometries studied is indicated in Figure 3.

**Figure 8:**
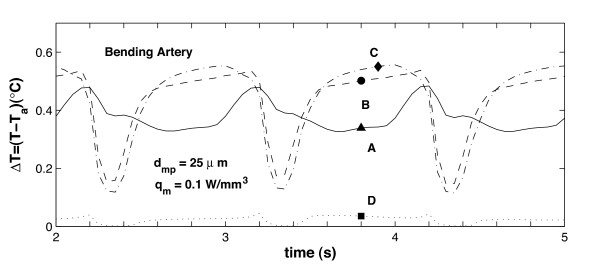
Variation over time of plaque/lumen temperature measured at four different points; the arterial geometry corresponds to the bending artery. The inlet velocity profile for this arterial geometry is given in Figure 2a, the points where the temperature is recorded correspond to points **A **through **D **shown in Figure 3.

**Figure 9:**
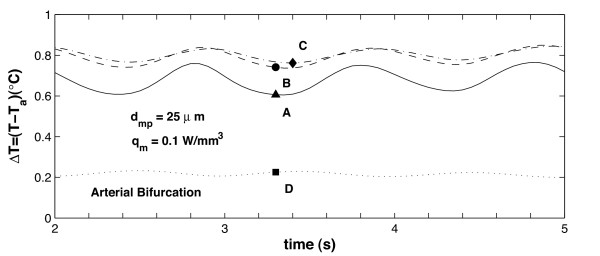
Variation over time of plaque/lumen temperature measured at four different points; the arterial geometry corresponds to the arterial bifurcation. The inlet velocity profile for this arterial geometry is given in Figure 2b, the points where the temperature is recorded correspond to points **A **through **D **shown in Figure 3.

Figures 8 and 9 show the transient temperature changes at points **A **through **D **for the bending artery and the arterial bifurcation, respectively. The temporal temperature changes registered during the cardiac cycle are between 0.1 and 0.6°*C *for the bending artery, and between 0.6 and 0.85°*C *for the arterial bifurcation. It is seen that the maximum temperature occurs at point **C**, which indicates the beginning of the rear region. In these figures, it is also noted that the temperature changes at points **B **and **C **follow the same trend during a cardiac cycle; at point **D**, the temperature remains relatively constant compared to the variation registered at the rest of the points. The separation flow and wake observed in the bending artery explain the relatively constant temperature at the end point, which means less thermal exchange by blood flow. For the arterial bifurcation (Figure 9) the same three thermal regions are observed, but the temperature at all the selected points (**A **through **D**) shows little variation with time, and the maximum temperature variation occurs at the mid point **A**.

**Figure 10:**
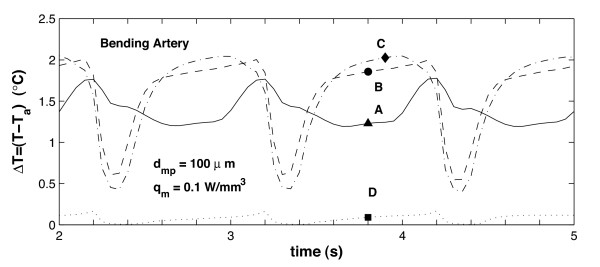
Variation over time of plaque/lumen temperature measured at four different points along the interface for the bending artery. This figure indicates temperature changes produced by variations in the macrophage thickness *d*_*mp*_. The inlet velocity profile for this arterial geometry is given in Figure 2a, the points where the temperature is recorded correspond to points **A **through **D**, located as indicated in Figure 3.

Comparison of Figures 8 and 10 shows that the maximum temporal temperature change in the bending artery ranges from 0.6°*C *to 2.0°*C *for *d*_*mp *_= 25 and 100 *μm*, respectively. The peak points of the maximum temporal temperature for *d*_*mp *_= 100 *μm *decreases to 0.5°*C *at *t *= 4.3 *s*, and increases to 2.0°*C *for *t *= 5.0 *s*. It is observed that as the macrophage layer thickness *d*_*mp *_increases, the maximum temperature Δ*T*_*max *_increases proportionally; this is identical to the behavior seen in the steady-state case [55] and follows the same linear trend observed experimentally [21,51].

**Figure 11:**
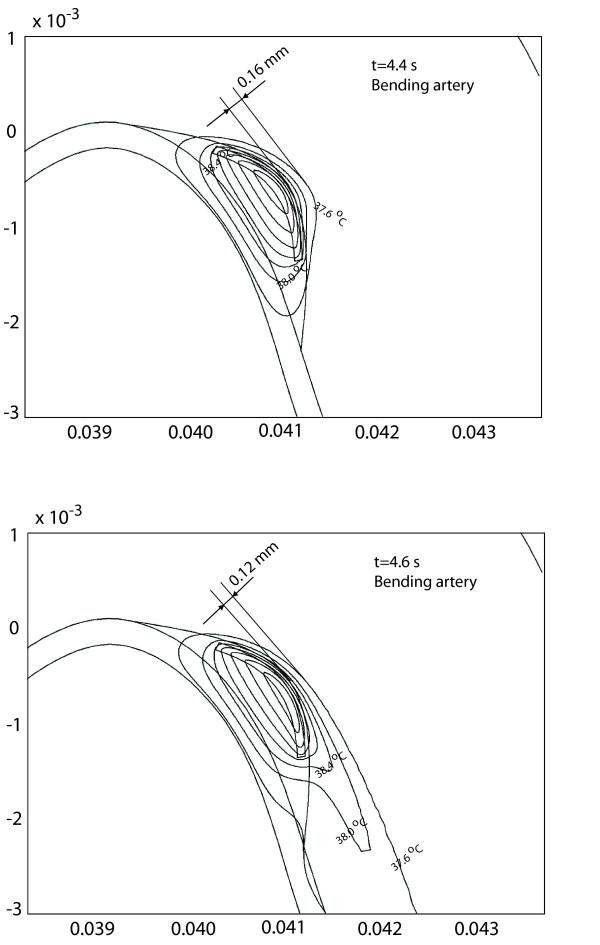
Temperature contours around an inflamed plaque located in a bending artery. Results shown correspond to two different times during the cardiac cycle: *t *= 4.4, and 4.6 *s*. Calculations were obtained using the inlet velocity profile of Figure 2a. In this figure, the macrophage layer dimensions are q˙
 MathType@MTEF@5@5@+=feaafiart1ev1aaatCvAUfKttLearuWrP9MDH5MBPbIqV92AaeXatLxBI9gBaebbnrfifHhDYfgasaacH8akY=wiFfYdH8Gipec8Eeeu0xXdbba9frFj0=OqFfea0dXdd9vqai=hGuQ8kuc9pgc9s8qqaq=dirpe0xb9q8qiLsFr0=vr0=vr0dc8meaabaqaciaacaGaaeqabaqabeGadaaakeaacuWGXbqCgaGaaaaa@2E20@_*m *_= 0.1 *W*/*mm*^3^, *l*_*mp *_= 1,500 *μm*, *d*_*mp *_= 100 *μm*, *d*_*p *_= 540 *μm*, and *l*_*f *_= 50 *μm*.

Figure 11 shows the temperature contours surrounding the atherosclerotic plaque in the bending artery for two different times during the cardiac cycle (*t *= 4.4 and 4.6 *s*). According to the inlet velocity profile of Figure 2, at *t *= 4.4 *s *the small forward flow has not reached the plaque; as a result, the temperature distribution is almost symmetric. The temperature of the blood surrounding the plaque only changes 0.1°*C *in a small region that does not extend beyond the macrophage layer length and separates up to 0.16 *mm *from the plaque surface. Meanwhile, the temperature contours at *t *= 4.6 *s *show a significant difference in the regions where the blood temperature surrounding the plaque is affected; such changes are correlated with the occurrence of flow separation. For *t *= 4.6 *s*, the temperature contour for *T *= 37.6°*C *separates from the plaque surface 0.12 *mm *at the plaque center, and the blood temperature variations are observed 4.5 *mm *from the plaque center to about 1 *mm *away from the plaque/lumen surface. Compared with the temporal temperature change at t = 4.4s, the blood temperature variations of 0.1°*C *are observed within 0.12 mm at the surface of plaque center during a cardiac cycle. The region where blood temperature changes are observed is a direct result of the plaque dimensions and metabolic heat production; such temperature changes, shown in Figure 11, correspond to the case of a macrophage layer with dimensions *l*_*mp *_= 1,500 *μm*, *d*_*p *_= 540 *μm*, *l*_*f *_= 50 *μm*, *d*_*mp *_= 100 *μm*, and a metabolic heat production of q˙
 MathType@MTEF@5@5@+=feaafiart1ev1aaatCvAUfKttLearuWrP9MDH5MBPbIqV92AaeXatLxBI9gBaebbnrfifHhDYfgasaacH8akY=wiFfYdH8Gipec8Eeeu0xXdbba9frFj0=OqFfea0dXdd9vqai=hGuQ8kuc9pgc9s8qqaq=dirpe0xb9q8qiLsFr0=vr0=vr0dc8meaabaqaciaacaGaaeqabaqabeGadaaakeaacuWGXbqCgaGaaaaa@2E20@_*m *_= 0.1 *W*/*mm*^3^.

Finally, from Figures 8, 9 and 11, it is seen that for a given arterial geometry, some locations at the plaque/lumen interface show significant temperature variations during the cardiac cycle. This observation is important to determine the sampling rate of the arterial wall temperature (AWT) measurements performed around the plaque region. In particular, it is seen that plaque temperature measurements should be performed in at least three locations; this is because temperature distributions are characterized by three thermal regions with different effects of convective and conductive heat transfer. Measurements should be taken during one or more cardiac cycles as temperature change is affected by the inlet pulsatile flow.

## Conclusion

The presence of the macrophage layer inside the plaque produces the appearance of a hot spot at the plaque/lumen interface. This work presents a mathematical model that can be used as a tool to improve direct arterial wall temperature measurements and the detection of vulnerable plaque. The location of the hot spot varies during the cardiac cycle and it is observed to be located behind the apex of the plaque. The exact location of the hot spot depends on the arterial geometry, the flow velocity, the presence of flow separation and recirculation, and the extension of the macrophage layer. The parameters that have the most influence over the plaque temperature are the macrophage layer thickness *d*_*mp *_and the metabolic heat accounting for the macrophage population and the inflammatory stage. The magnitude of q˙
 MathType@MTEF@5@5@+=feaafiart1ev1aaatCvAUfKttLearuWrP9MDH5MBPbIqV92AaeXatLxBI9gBaebbnrfifHhDYfgasaacH8akY=wiFfYdH8Gipec8Eeeu0xXdbba9frFj0=OqFfea0dXdd9vqai=hGuQ8kuc9pgc9s8qqaq=dirpe0xb9q8qiLsFr0=vr0=vr0dc8meaabaqaciaacaGaaeqabaqabeGadaaakeaacuWGXbqCgaGaaaaa@2E20@_*m *_is assigned the values 0.05,0.1 and 0.2 *W*/*mm*^3 ^which produces a maximum temperature change comparable with the reported measurements [21,51]. The calculations reproduce the linear relationships observed experimentally between the macrophage layer thickness *d*_*mp *_and the maximum plaque temperature Δ*T*_*max*_.

It is observed that the best location to measure plaque temperature in the presence of blood flow is between the middle and the far edge of the plaque, as the highest spatial temperature variation occurs within this region; particularly, the maximum temperature location occurs between between 0.5 <*l*/*l*_*p *_< 0.7 for the bending artery, and is steadily located at *l*/*l*_*p *_= 0.7 for the arterial bifurcation model. It is seen that the maximum spatial temperature location travels within 0.6 *mm *along the longitudinal direction for the bending artery during one cardiac cycle. Compared with the spatial resolution of 0.5 *mm *of thermography in specially designed catheters [12,22,24], the path of the maximum spatial temperature location can not be ignored while directly measuring AWT during the cardiac cycles. Considering the temporal temperature change calculated for the bending artery, direct measurement of arterial wall temperature should be taken very close to the plaque/lumen surface; the blood temperature variations of 0.1°*C *are observed within 120 *μm *from the surface of the plaque center during a cardiac cycle. It is seen that the transient temperature variation at the plaque/lumen interface is different depending on the arterial geometry and the velocity waveform; finally, in order to have representative local temperature measurements, the temperature should be recorded in the same location over at least one full cardiac cycle.

The model proposed herein can be used to analyze the cooling effect of blood over the plaque surface and the effect of plaque geometry, size, and location in the artery. However, to improve the predictive nature of the model, it is necessary to consider four significant improvements: **(1) **realistic geometry, **(2) **the presence of turbulence, **(3) **non-newtonian characteristics of blood, and **(4) **Fluid-Structure interaction.

The current work employs idealized geometries derived from anatomical descriptions. This approximation is performed because realistic geometries are exceedingly complex due to the irregular shape and heterogeneous structure of atherosclerotic plaque. The geometries in this study assume a constant diameter, a smooth and round luminal surface, and a smooth boundary between the plaque and macrophage layers. Future studies should contemplate three dimensional and realistic geometries, to estimate the predictive nature of the model against direct measurements, as well as the need for realistic geometry to analyze the factors affecting arterial wall temperature.

The presence of turbulence can induce a large impact for temperature distributions which may be detected near plaques; it is expected that this effect is larger in plaques located by the arterial bifurcation or segments where large curvatures are detected. This study shows that the effect of turbulence depends on the arterial geometry and the velocity waveform. Turbulence profoundly influences the mechanisms of heat and momentum transfer in a thermal boundary layer, so future studies should introduce different turbulence models [56].

Multi-phase systems need to be adopted to minimize the potential errors which arise from neglecting the variation of blood viscosity. The theory indicates heat transfer in blood-solid flow with components (*i.e. *erythrocytes, plasma, etc.) is influenced by the momentum transport process. The plaque regions with low Reynolds numbers could show a denser packing of blood particles by diffusion-dominated flow. Such dense blood particles appreciably affect the convective heat transfer between both blood flow and particle components. However, blood flow (assumed as Newtonian fluid) in large arteries can be a dilute flow which keeps a sufficiently large distance between individual particles in order to avoid significant particle-particle interactions [57]. Finally, this study neglects the interaction between the fluids and structures which relate to the deformation of the arterial wall as well as the stress distribution over the plaque. Because of the hot spot formation due to the inflammation experienced by the plaque, consideration of the fluid-structure interaction and the calculation of thermal stresses produced by the hot plaque seems to be the most important characteristics missing from the proposed model.

In future studies, this model will be used to estimate the errors associated to direct measurements of AWT using the basket catheters described in [23,24]; these catheters are formed by an array of thermocouples and are used to measure the temperature alterations in the arterial wall due to plaque activity. When catheters are introduced, the flow is disturbed, reduced, or completely occluded. This in turn should affect the temperature at the plaque surface. The present model used to analyze the alterations in AWT during the blood flow interruption or reduction may further elucidate the convective cooling effect of blood flow during measurement using catheters.

## Competing interests

The author(s) declare that they have no competing interests.

## Authors' contributions

Over 13.2 million Americans are affected by coronary heart disease and unpredicted heart attacks produced by inflamed plaque account for the majority of the 280 billion dollar burden of cardiovascular disease. Many engineers study flow in arteries and this has lead to understanding how the location of plaque and the effects of fluid forces influence stability. Alternately, the authors intend to understand plaque evolution through plaque temperature because Arterial Wall Thermography (AWT) can help detect plaque vulnerability. In addition, clinical research groups experimentally studying AWT have divergent opinions about the cooling effect of blood and other parameters on temperature measurement. This work addresses some of these factors using a mathematical model and parametric studies.
